# Assessing the risk of foreign investment within the petroleum sector of South America

**DOI:** 10.1007/s43546-022-00221-6

**Published:** 2022-05-20

**Authors:** Yeltsin Tafur, Eric Lilford, Roberto F. Aguilera

**Affiliations:** 1 Department of Mineral and Energy Economics, Curtin University, Perth, WA Australia; 2 Curtin University Oil and Gas Innovation Centre (CUOGIC), Perth, WA Australia

**Keywords:** Fiscal regimes, Government take, International capital flow, Oil revenues, Petroleum taxation, Proven oil reserves, Q30, Q32, Q35, Q38

## Abstract

There is presently a shortage of international oil companies investing in South America, due primarily to political instability associated with high levels of corruption, poor quality of institutions, and demanding fiscal regimes that strip significant amounts of revenue from investors. The purpose of this research is to obtain a comprehensive country ranking for South America in terms of investment risk in the upstream oil sector. The study identifies six risk categories (political risk, macroeconomic risk, technical risk, investment climate, non-renewable energy resources potential, and environmental constraint) and ten sub-indicators associated with these risks. The data are gathered to perform an ‘analytic hierarchy process (AHP)’ to obtain the weight index of the ten sub-indicators. These are then used in a ‘technique for order preference by similarity to ideal solution (TOPSIS)’ to obtain the country-ranking risk arrangement. Results indicate that countries with low-risk investment include Brazil, Colombia and Peru, while high-risk countries include Argentina, Ecuador and Bolivia. Finally, this study suggests that countries whose proportions of government take exceed 75% should modify their fiscal regimes to optimize benefits for all parties or design fiscal systems where the host government and contractor share the risk and reward associated with exploiting oil resources.

## Introduction

Upstream oil activities are exposed to economic and financial risks on account of significant capital investments, technical risks associated with availability of technology and workforce skills, the amount of proven oil reserves, climate policies for low-carbon economic development, and political risks (Duan et al. 2018). There is a shortage of international oil companies (IOCs) operating in developing countries, as these countries are often viewed as unfavorable destinations in which to invest (Jude and Levieuge 2017).

The collapse of oil demand as a result of the COVID-19 pandemic, combined with geopolitical factors, has caused a profound negative impact for oil-producing countries; e.g., significant reductions of their national budgets and societal needs. For oil exploration and production (E&P) companies, this turmoil has caused the delay of new projects, slashed their expenditures at existing operations, and collapsed their net cash flows.

The risk assessment of upstream oil projects will be different in future decades due to the transition to low-carbon energy (McCauley et al. 2019) and public health concerns that affect the demand and supply of non-renewable, carbon-rich commodities. The macroeconomic framework driven by fiscal regimes in developing resource-rich countries (RRCs) will be key to promoting the development of their natural resources and the final investment decisions of international firms (Daniel et al. 2017).

The vast majority of developing countries in South America are highly dependent on the exploitation of natural resources, including metals, minerals and petroleum (Ossowski and Halland 2016). In countries such as Ecuador and Venezuela, the contributions from petroleum revenues in the form of royalties and taxes represents 25 and 50% of fiscal income, respectively (Cameron and Stanley 2017). Within South America, national oil companies (NOCs) are the ones that have the highest levels of participation in exploration and exploitation (Berrios et al. 2011). On the other hand, IOCs fear being nationalized due to some of the past cases that have occurred in most countries of the region (Mahdavi 2014).

Energy security has been a serious concern in emerging markets and low-income countries across South America, where energy demand has been continuously increasing due to population growth and economic development (Wolfram et al. 2012). Oil import risk is another concern, with policymakers emphasizing the importance of reduced external oil dependency by enhancing domestic energy availability and improving energy efficiency (Mohsin et al. 2018). As South American countries have been economically weakened by the COVID-19 pandemic, energy cooperation in the region and promotion of its extractive natural resources are seen as vital to support the post-pandemic economic recovery. However, given the intensified pressure in recent years to reduce emissions and other environmental and social impacts, development of petroleum is likely to be dependent on the mitigation of such issues.

There are various methodologies available for calculating the risk of foreign direct investment (FDI), such as real-option analysis and sensitivity analysis (Fan and Zhu 2010). This study uses methodologies of multiple attribute decision making (MADM) and employs the analytical hierarchy process (AHP) to determine the weight index of attributes associated with the risk of investment. Moreover, it uses the technique for order preference by similarity to ideal solution (TOPSIS) to obtain the comprehensive country ranking, from low- to high risk, for petroleum-producing countries in South America. However, there are countries in the region that are being excluded from this research due to high levels of economic and political instability, a lack of data (e.g., Venezuela), and shortages of petroleum resources (e.g., Chile, Paraguay, and Uruguay) (IEA 2020). The chosen methodologies are well established in the literature; e.g., they were used to assess foreign oil investments by Chinese petroleum companies (Li et al. 2016), and to analyze the petroleum investment environment in Asia (Duan et al. 2018).

The present study is original in that it assesses the risks of international capital flows into South America by utilizing AHP and TOPSIS methodologies to determine a country ranking of foreign oil investments. As such, it is intended as a tool to support IOCs in their final investment decisions in the region. Furthermore, the study can assist policymakers when reassessing their petroleum fiscal regimes to enhance investment attractiveness.

 The rest of this work is structured according to the following main sections: “Theoretical Framework”; “Risk Categories and Sub-Indicators for Overseas Oil Investments”; “Research Design”; “Results and Analysis”; “Policy Recommendations”; and “Conclusions”.

## Theoretical framework

This section starts with a review of the existing literature on FDI in the oil sector and then describes the risk assessment methods to be used, as well as the AHP and TOPSIS methodologies. In addition, it includes a brief description of the foreign oil investment experience in South America.

### Literature review

Previous research assessed the risk of FDI within the resources and petroleum sectors. The risks for international companies are based on a high degree of uncertainty and complexity—influenced by commodity prices, geopolitical factors, and political and economic instability. In addition, the macroeconomic framework driven by fiscal systems and petroleum taxation help to explain the flexibility of fiscal rules during commodity booms and recessions.

There are many determinants to consider in an FDI destination. A wide variety of literature analyzes exogenous factors (e.g., taxes, exchange rates) and how these factors affect the final decisions of investors (Blonigen 2005). Using AHP and TOPSIS, Li et al. (2016) assessed the risk of FDI in the shale gas sector for Chinese oil companies. In addition, it identified five categories of risk, including economic, political, geological, technological, and internal management risk. Duan et al. (2018) applied a fuzzy integrated model based on entropy weight to review China’s Belt and Road Initiative. The aim was to evaluate energy security and energy investment risks for China. The study found that resource potential and diplomatic factors are the main determinants for investing in energy projects. In a qualitative–quantitative comprehensive risk evaluation method to analyze FDI in oil refining projects for Chinese oil and gas companies, Li et al. (2017) identified the following risk factors: investment environment risk; organization management risk; technical risk; health, safety, environmental and social responsibility risk; and economic risk. Meanwhile, Tian et al. (2020) conducted a study using AHP and TOPSIS that focused on investment in arable land resources through China’s Belt and Road Initiative. They concluded that Chinese companies tend to invest in countries that have ample cultivable resources and low corruption indices.

The present work aims to determine a country ranking, from low- to high risk, of foreign oil investment in South America by employing the AHP and TOPSIS methodologies. It is intended to assist IOCs in making final decisions about favorable destinations for investment.

### Multiple criteria decision-making

**Fig. 1:**
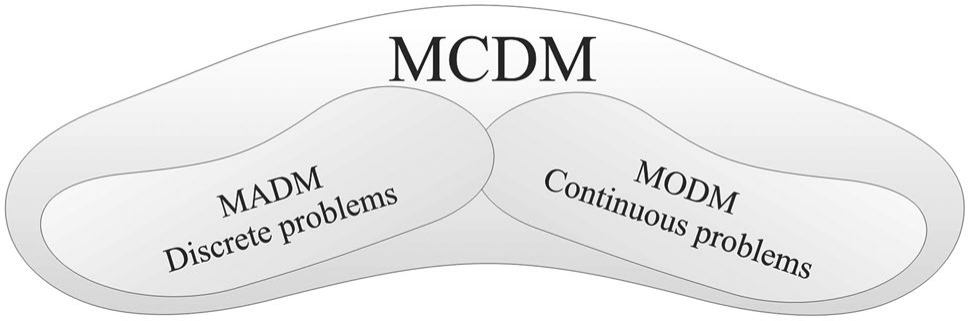
Two Types of MCDM. Source: Zavadskas et al. (2014)

Multiple criteria decision-making (MCDM) is a method used to make decisions that involve many criteria and sub-criteria (Byun and Lee 2006). It can be classified into two categories: multiple attribute decision making (MADM) and multiple objective decision-making (MODM). MADM methods are employed to solve discrete problems that involve selection from a finite number of options. On the other hand, MODM methods have decision variables that are determined to solve continuous problems with either an infinite or a large number of choices (Rao 2007). Figure 1 depicts the two categories of MCDM.

This study employs a finite number of options or sub-indicators for building a comprehensive decision matrix to determine the country-ranking model using AHP and TOPSIS methodologies, which are part of MADM.

MADM searches for the best among existing 'actions', 'candidates', 'options', 'policies' or 'alternatives' by considering multiple 'criteria', 'attributes', or 'objectives' that are in conflict with each other. It resolves the decision making with a table or matrix, as shown in Table 1. It then calculates the weight index based on the relative importance from Table 2 (Kuo et al. 2008; Zanakis et al. 1998). The most common methodologies for MADM are the weighted product method (WPM), analytic hierarchy process (AHP), and the technique for order preference by similarity to ideal solution (TOPSIS) (Chen and Hwang 1992; Rao 2007). The AHP method is applied in this paper to obtain the weight coefficients that denote the relative importance of the sub-indicators, which are then used in TOPSIS to obtain the country ranking.

**Table 1:** Decision table in MADM methods

Alternatives	Attributes					
	B_1_ (w_1_)	B_2_ (w_2_)	B_3_ (w_3_)	– (–)	– (–)	B_M_ (w_M_)
A_1_	m_11_	m_12_	m_13_	–	–	m_1M_
A_2_	m_21_	m_22_	m_23_	–	–	m_2M_
A_3_	m_31_	m_32_	m_33_	–	–	m_3M_
–	–	–	–	–	–	–
–	–	–	–	–	–	–
A_N_	m_N1_	m_N2_	m_N3_	–	–	m_NM_

Source: Rao (2007)

**Table 2:** Scale of preference between two attributes or indicators in AHP

Preference factor	Degree of preference	Explanation
1	Equally	Two factors contribute equally to the objective
3	Moderately	Experience and judgment slightly to moderately favor one factor over another
5	Strongly	Experience and judgment strongly or essentially favor one factor over another
7	Very strongly	A factor is strongly favored over another and its dominance is showed in practice
9	Extremely	The evidence of favoring one factor over another is of the highest degree possible of an affirmation
2, 4, 6, 8	Intermediate	Used to represent compromises between the preferences in weights 1, 3, 5, 7 and 9
Reciprocals	Opposites	Used for inverse comparison

Source: Saaty (2008)

The decision matrix in MADM has four parts: (1) alternatives; (2) attributes; (3) relative importance of each attribute (i.e., weight index); and (4) measures of performance of alternatives with respect to the attributes.

### Analytic hierarchy process (AHP)

AHP is considered the most useful tool for solving decision-making problems (Saaty 2000). Saaty (1988) developed this method and broke a decision-making problem down into a system of hierarchies of objectives, attributes (or criteria) and alternatives.

The main procedure of AHP employs the geometric mean method (Rao 2007), consisting of the following steps:

**Step 1:** Determine the objective to perform decision making and evaluation of attributes to achieve this goal. The goal is entered at the top level, the attributes at the second level, and the alternatives at the third level.

**Fig. 2:**
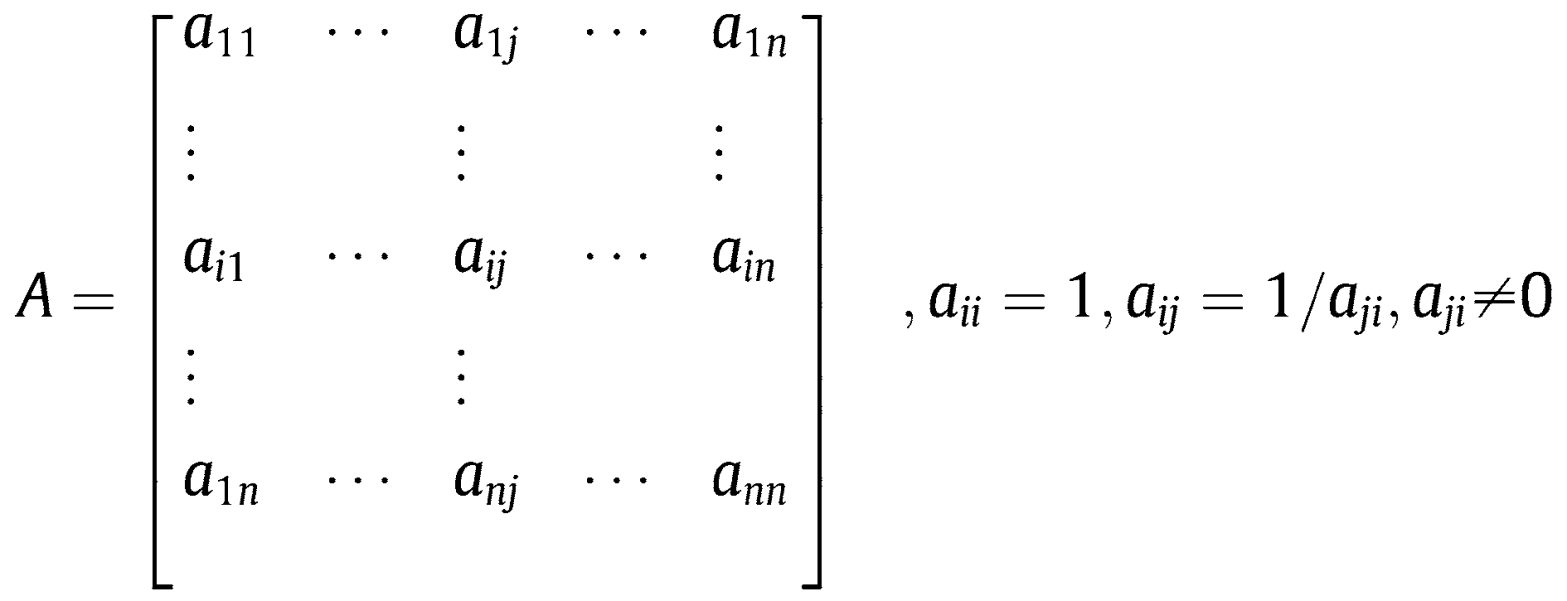
Pairwise comparison matrix. Source: Çalışkan et al. (2013) ; Rao (2007)

**Step 2:** Find the relative importance of different attributes with respect to the goal. The pairwise comparison matrix from Fig. 2 (where the criteria are denoted by *a*_1_, *a*_2_, …, *a*_*n*_) is built using the scale of importance shown in Table 2.

The relative importance of two criteria is rated using a scale of preference from Table 2 (Saaty 2008) with the values 1 (equally important), 3 (slightly more important), 5 (much more important), 7 (demonstrably more important) and 9 (absolutely more important). The values 2, 4, 6 and 8 are used to facilitate a compromise between slightly differing judgments (Çalışkan et al. 2013)

**Step 3**: Calculate the relative normalized weight (wj) of each attribute by calculating the geometric mean (GM).

1 $${\mathrm{GM}}_{j}={\left[{\prod_{j=1}^{M}}{b}_{ij}\right]}^{1/M} ,$$

2 $${w}_{j}=\frac{G{M}_{j}}{{\sum }_{j=1}^{M}GMj} .$$

Calculate matrices A3 and A4 such that A3 = A1 × A2 and A4 = A3/A2, where A2 = [*w*_1_, *w*_2_, …, *w*_j_]^*T*^.

The comparative weights of the coefficients from matrix A2 are obtained by finding the eigenvector $w$ with respective to ${\lambda }_{\mathrm{max}}$ that satisfies *Aw* = ${\lambda }_{\mathrm{max}}w$, where ${\lambda }_{\mathrm{max}}$ is the largest eigenvalue of pairwise comparison matrix A (Çalışkan et al. 2013).

To ensure the consistency of the subjective perception and the accuracy of the comparative weights, the consistency index (CI) and the consistency ratio (CR) are calculated. The formula for the CI is defined as follows:

3 $${\text{CI}}\, = \,( \lambda_{\max } {-}M)/\left( {M{-}1} \right).$$

Table 3 shows the average random index (RI) values proposed by Saaty (1988) for the number of attributes or size matrices analyzed that help to determine CR.

**Table 3:** Random index values

Attributes	3	4	5	6	7	8	9	10
Random Index (RI)	0.52	0.89	1.11	1.25	1.35	1.4	1.45	1.49

Source: Saaty (1988) and Rao (2007)

The CR is obtained by comparing the CI with the appropriate value from Table 3 (Saaty 1988), and it is defined as follows:

4 $${\text{CR}}\, = \,{\text{CI}}/{\text{RI}}.$$

The CR should be under 0.1 for a reliable and acceptable result (Tzeng and Huang 2011). An inconsistency of 0.1 or less implies that the adjustment is small compared to the actual values of the eigenvector entries (Saaty and Vargas 2012).

**Step 4:** The next step is to obtain the overall or composite performance scores for the alternatives by multiplying the relative normalized weight (*w*_j_) of each attribute (obtained in step 2) with its corresponding normalized weight value for each alternative (obtained in step 3).

### Technique of order preference similarity to the ideal solution (TOPSIS)

The TOPSIS method was proposed and developed by Hwang and Yoon (1981). It is based on the concept that the chosen alternatives should have the shortest Euclidean distance from the ideal solution and the farthest from the negative ideal solution. Moreover, it requires information on the relative importance of properties that are considered in the selection process.

The TOPSIS method consists of the following steps:

**Step 1:** The normalized decision matrix, *R*_*ij*_, is defined as follows:

5 $${R}_{ij}=\frac{{m}_{ij}}{{\left[\sum_{j=1}^{M}{m}_{ij}^{2}\right]}^{{1}/2}} ,$$

where *R*_*ij*_ denotes the normalized value of the *j*th criterion for the *i*th alternative *Ai*.

**Step 2:** Calculate the weighted normalized decision matrix:

6 $$V_{ij} \, = \,w_{j} \, R_{ij} , \, i\, = \,1, \, \ldots , \, M; \, j\, = \,1, \, \ldots , \, n,$$

where *w*_*ij*_ is the weight of the *j*th criterion or attribute.

**Step 3:** Determine the positive ideal and negative ideal solutions, which are defined as follows:

7 $${V^ + } = \left\{ {\mathop {\left( {\sum\limits_i {{v_{ij}}} /j \in J} \right)}\limits^{\max } ,\mathop {\left( {\sum\limits_i {{v_{ij}}} /j \in J'} \right)}\limits^{\min } ,i = 1,2, \ldots ,N} \right\} = \left\{ {V_1^ + ,V_2^ + ,V_3^ + , \ldots ,V_M^ + } \right\},$$

8 $${V^ - } = \left\{ {\mathop {\left( {\sum\limits_i {{v_{ij}}} /j \in J} \right)}\limits^{\min } ,\mathop {\left( {\sum\limits_b {{v_{ij}}} /j \in J'} \right)}\limits^{\max } ,i = 1,2, \ldots ,N} \right\}, = \left\{ {V_1^ - ,V_2^ - ,V_3^ - , \ldots ,V_M^ - } \right\},$$

where *V*^+^ denotes the positive ideal solution, and *V*^−^ the negative ideal solution. If the *j*th criterion is a beneficial criterion, then *v*_*j*_^+^  = max {*v*_*ij*_, *i* = 1, …, *M*} and *v*_*j*_^−^ = min {*v*_*ij*_, *i* = 1, …, *M*}. In contrast, if the *j*th criterion is a beneficial criterion, then *v*_*j*_^+^  = min {*v*_*ij*_, *i* = 1, …, *M*} and *v*_*j*_^−^ = max {*v*_*ij*_, *i* = 1, …, *M*}.

**Step 4:** Calculate the distances from each alternative to a positive ideal solution and a negative ideal solution:

9 $${S}_{i}^{+}=\sqrt{\sum_{j=1}^{M}{\left({v}_{ij}-{v}_{j}^{+}\right)}^{2}}, i=1,\dots ,N,$$

10 $${S}_{i}^{-}=\sqrt{\sum_{j=1}^{M}{\left({v}_{ij}-{v}_{j}^{-}\right)}^{2}}, i=1,\dots ,N,$$

where *S*_i_^+^ denotes the distance between the *i*th alternative and the positive ideal solution, and *S*_i_^−^ denotes the distance between the *i*th alternative and the negative ideal solution.

**Step 5:** Calculate relative closeness to the ideal solution.

11 $${P}_{i}={S}_{i}^{-}/\left({S}_{i}^{+}+ {S}_{i}^{-}\right).$$

**Step 6:** Rank the alternatives, sorting by the performance score values (*P*_*i*_) in decreasing order. The higher values of *P*_*i*_ mean that the rank is better.

### Foreign oil investments

FDI in the upstream oil sector is crucial for economic development in lower income countries with abundant oil resources (Guilford et al. 2011). This brings broad benefits such as capital, knowledge, skills, technology, and employment (Bayulgen 2010). However, FDI is generally limited in the oil industries of developing nations—while some have at times managed to create attractive investment regimes, others have failed despite having sound macroeconomic conditions (Addison and Roe 2018). Why do some countries have investor-friendly policies and others not? Do domestic institutions affect the attractiveness of foreign oil investments? The answers are that a lack of transparency in the domestic institutions of many developing countries, based on particular political interests, puts them at a disadvantage for attracting FDI (Bayulgen 2010; Jensen 2008).

IOCs have concerns about investing in host countries where there could be interference through nationalizations or new regulatory requirements (Bayulgen 2010). South America has a history of extractive industries (EIs) nationalism in which the host country takes possession of petroleum or mining projects (Berrios et al. 2011). Examples of this can be found in Argentina, Bolivia, Peru, Ecuador, and Venezuela (Pierce 2011). In May 2006, Bolivia nationalized its oil and gas industry after the president, Evo Morales, assumed his mandate, with increased participation of the state oil company thereafter. In the same month and year, Ecuador took over the installations of Occidental Petroleum, which then became part of state-owned Petroecuador. In December 2006, Venezuela forced the exit of Exxon and ConocoPhillips, while other IOCs (such as BP and Statoil) were forced to reduce their participation in the Venezuelan petroleum industry (Click and Weiner 2010). Table 4 shows the history of oil and gas nationalization in Latin America.

**Table 4:** Nationalization in the petroleum industry of Latin America

Country	Year of nationalization
Argentina	1922, 1924, 1930, 2004
Bolivia	1937, 1969, 2006
Brazil	1953
Chile	1932, 1950
Colombia	1951
Ecuador	1972, 1974, 2006
Mexico	1938
Peru	1968, 1986
Uruguay	1931
Venezuela	1976, 2001

Source: Berrios et al. (2011)

## Risk categories and sub-indicators for overseas oil investments

This section describes and identifies the main categories of risks and sub-indicators associated with the upstream oil industry, as well as the fiscal regimes and instruments, in order to give a comprehensive perspective of overseas oil investments.

**Fig. 3:**
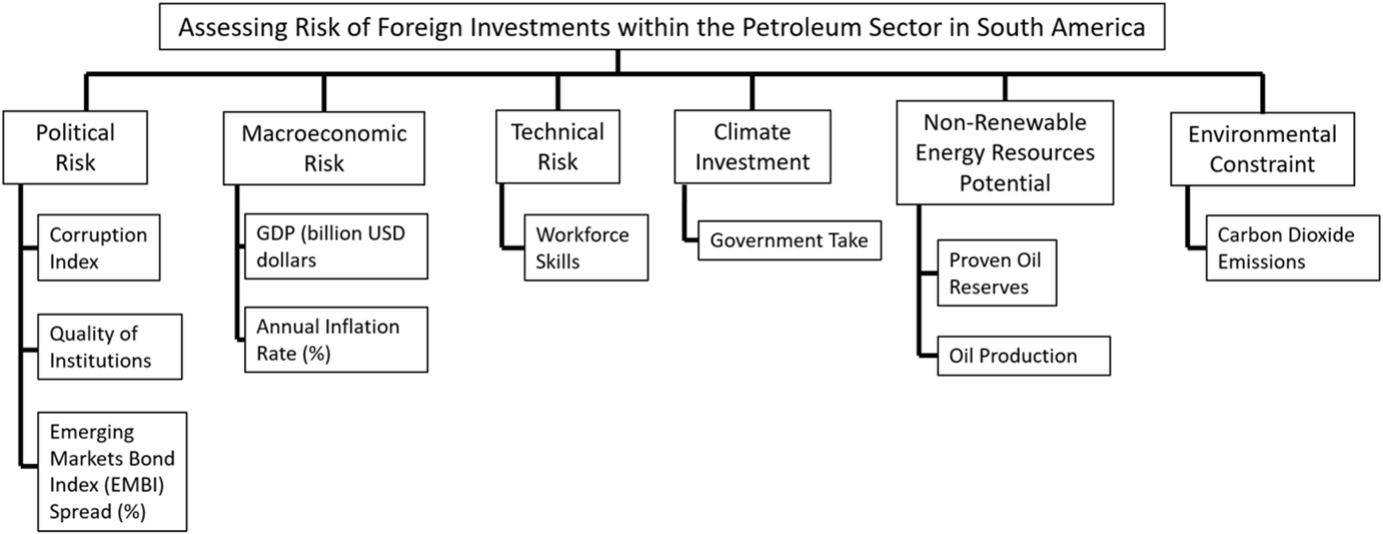
Risk categories and sub-indicators for upstream oil investments

The six risk categories and ten sub-indicators shown in Fig. 3 are used to develop the AHP and TOPSIS methods, which determine a comprehensive country ranking of foreign oil investments in South America.

### Political risk

Before the Cuban revolution of 1953–1959, FDI in Latin America from the United States was approximately USD 338 million. After the revolution, this figure fell to USD 95 million in 1960 and the net flows turned negative in 1962. One of the main reasons that FDI diminished was the political crisis that was spreading throughout Latin America (Levis 1979). Busse and Hefeker (2007) sampled 83 developing countries from 1984 to 2003 and found the following indicators to be detrimental on FDI: government instability; internal and external conflict; corruption; and quality of institutions (bureaucracy). The latter two, plus an ‘emerging market bond index (EMBI)’, are discussed next.

#### Corruption index

According to Gupta and Abed (2002), corruption is defined as the abuse of entrusted power for private gain**,** and it is a feature of low-income countries that affects economic development. The World Economic Forum, in its Global Competitiveness Report (GCR) for 2019, compiled a ranking of 141 countries based on a computed corruption index (0–100). It gave the highest points for good transparency (e.g., Denmark) and the lowest points for the worst transparency (e.g., Venezuela).

#### Quality institutions

Having efficient institutions is key to designing and implementing sound economic policies that result in reliable and high-quality public reporting data on government finances—critical to effective fiscal management and accountability (Alt et al. 2006; Heald 2003). In the first ‘pillar’ of the GCR, institutions, there is a sub-section: public-sector performance, which translates to countries having a high score if they possess high-quality institutions.

#### Emerging markets bond index (EMBI)

The EMBI spread is a debt benchmark index, proposed by JP Morgan, which measures the total performance of government and corporate bonds issued by emerging market countries that meet specific liquidity and structural requirements. Having a high EMBI spread means that the country presents a high risk for investment (e.g., Venezuela, Argentina and Ecuador, in the South American context).

### Macroeconomic risk

The macroeconomics of natural resources can be divided into three interconnected areas: commodities markets, growth in commodity exporters, and economic diversification (Bova et al. 2018; Davis 1992). This study uses macroeconomic risk indicators including the gross domestic product (GDP) and annual inflation rate. GDP is an economic indicator that measures the total market value of all finished goods and services produced within a country (Kravis et al. 1975). Encinas-Ferrer (2015) pointed out that an increase in GDP attracts FDI. The inflation rate is calculated as the annual percentage change in the consumer price index (CPI) (Bruno and Easterly 1998). Singhania and Gupta (2011) stated that the inflation rate has a strong effect on economic growth and it is a determinant of FDI inflows because it influences the final rates of returns on investment.

### Technical risk

Technical risks appear at the start of the development of mining or petroleum projects, including risks in the technical design, commissioning and operations (Li et al. 2016). This study focuses on human capital skills as risks of FDI and takes into account workforce skills as a technical risk indicator.

Natural extractive resource projects require a skilled and trained workforce to meet the demand of energy and mineral resources (McKenzie and Hoath 2014; Saxinger 2016). Skilled labor shortages have become a barrier to developing petroleum and mining projects due to the remote locations of the natural resources (Storey 2010; Tonts 2010). For this reason, natural resource companies are compelled to fill the shortage of qualified workers by employing non-resident workers who are typically rostered on a fly-in, fly-out (FIFO) or drive-in, drive-out (DIDO) basis (Carrington and Pereira 2011).

Meeting the demand with a local qualified workforce at the beginning of the petroleum project would help the IOC save on its foreign investment budget. However, there are shortages of qualified local personnel in South America, when compared to North America and the Middle East (Gugler and Brunner 2007).

### Climate investment

The local climate investment risk refers to rigid policies causing the disruption of non-renewable resource projects, which creates social and environmental issues (Jiang and Sinton 2011). Johnston (2018) indicates that government take (GT) is a proportion of the government’s share of economic profits from petroleum and mining activities. There are four main ways for the host government to collect resources revenues and to benefit financially: royalties; profit-based mechanisms (e.g., taxes, profit oil sharing); government participation; and bonuses.

In all cases, the host government will aim to maximize the GT through the design of its fiscal regime; i.e., royalties and taxes (Luca and Mesa 2016). RRCs are required to develop an overall fiscal mechanism that optimizes GT while encouraging FDI.

### Non-renewable energy resource potential

Physical resource potential within a perspective country is one of the key indicators for determining the viability of petroleum foreign investments (Fan and Zhu 2010). RRCs attract foreign investments that eventually generate resource revenues (Berg et al. 2013). This study takes into account the following resource potential indicators: proven oil reserves and oil production.

Data from BP (2020) and IEA (2020) indicates that South America accounts for 18.7% of the total proven oil reserves in the world, while Venezuela has the world’s largest proven oil reserves at 303.3 billion barrels. The price of oil and the cost of capital will have an impact on the discovery of new oil reserves (Ewing 2017). One of the findings of this paper is that IOCs look for countries that have significant amounts of proven oil reserves; these will allow for sufficient production to compensate for payments made to the host government.

BP (2020) states that the total oil production share in South America represents 6.5% of the world total, from which Brazil (the largest producer in the region) represents 3.0% at 2.9 million b/d.

### Environmental constraint

The context of the energy market is changing for oil companies, oil-exporting countries and societies around the world based on three main factors: climate change, technology and societal expectations (Fattouh et al. 2019). This study considers that it is important to evaluate the push for of low-carbon economic development (promoting renewable energy projects and discouraging petroleum projects).

**Fig. 4:**
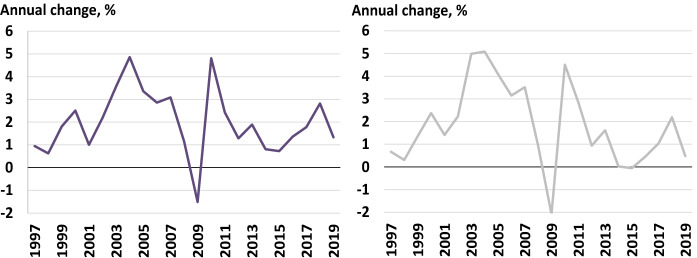
Profiles of primary energy consumption (left) and carbon emissions (right). Source: BP (2020)

In the coming decades, oil is expected to continue to represent an important part of primary energy consumption (Behera and Dash 2017). For this reason, FDI within the upstream oil sector needs to justify the viability of climate policies and carbon budgets dictated by the host government (Jaccard et al. 2018). This study considers CO_2_ emissions as a sub-indicator that helps RRCs in promoting their upstream oil sector under the global carbon budget that would prevent a 2 °C global temperature rise. Figure 4 depicts the growth in primary energy consumption on the left and CO_2_ emissions from energy use on the right.

## Research design

**Fig. 5:**
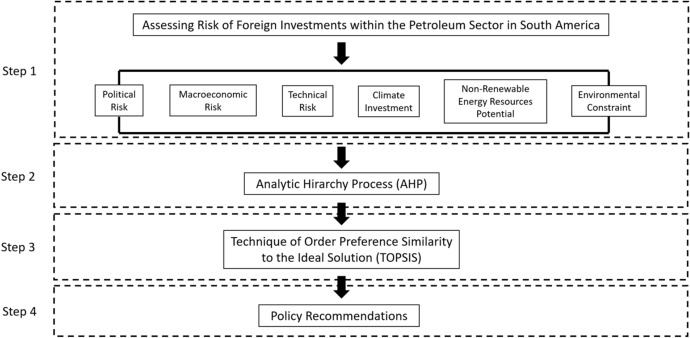
Methodology flowchart

Figure 5 depicts the methodology flowchart employed in this study, which is comprised of four steps. First, six categories of risk investments are identified in relation to the exploration and exploitation of non-renewable energy resources. Second, the AHP is applied to determine the weight index of ten sub-indicators. Third, the TOPSIS method is performed to rank countries based on the AHP weight index results. Fourth, policy recommendations for the host government and investor perspectives are provided to promote FDI.

This section details the procedure to determine the country ranking from low- to high risk of overseas oil investments in South America, which has been subdivided into three parts: (1) collection and processing of the ten sub-indicators used in the model for the six countries evaluated in the model; (2) elaboration of the pairwise comparison matrix with the ten sub-indicators and calculation of the criteria weights of each; and (3) application of TOPSIS to obtain the ranking of countries.

### Gathering and processing information

The six risk categories and ten sub-indicators associated with the upstream oil sector were collected and processed from open sources. Table 5 shows the main data and information sources for this study, between 2019 and 2020.

**Table 5:** Main sources for gathering information

Risk categories	Sub-indicators	Data sources
Macroeconomic risk	GDP (billion USD dollars)	IMF and World Bank
	Annual Inflation Rate (%)	IMF and World Bank
Climate investment	Government Take	IMF
Political risk	Corruption Index	GCR
	Quality of Institutions	GCR
	Emerging Markets Bond Index (EMBI) Spread (%)	Bloomberg and Reuters
Non-renewable energy resources potential	Proven oil reserves	BP Statistical Review and EIA
	Oil Production	BP Statistical Review and EIA
Technical risk	Workforce Skills	GCR
Environmental constraint	Carbon Dioxide Emissions	EIA and World Bank

#### Country selection

South America is a petroleum and mining resource-rich continent composed of twelve countries. However, the criteria for choosing countries in this study is based on their oil resource potential and government take. The former encourages overseas oil investments based on the potential for oil production, and the latter helps to understand the fiscal regimes—crucial for estimating the contractor take, or investor revenue, of petroleum production.

This study does not consider countries that have meagre oil resource potential or are net importers of oil, such as Chile, Paraguay, Uruguay, Suriname and Guyana (BP 2020). Furthermore, Venezuela is not part of this analysis due to the scant and inaccurate information for GDP, annual inflation rate, government take, corruption index, quality institutions, EMBI and CO_2_ emissions. Its current political and economic instability makes Venezuela a risky place to invest within the upstream oil sector (Schwab 2020).

As a result, this research considers six petroleum-producing countries in South America to perform the country-ranking risk arrangement for overseas oil investments: Argentina, Bolivia, Brazil, Colombia, Ecuador and Peru (ordered alphabetically).

### Weighting index of sub-indicators

At this stage, the pairwise comparison matrix is elaborated using the ten sub-indicators or attributes from Table 5. A scale of relative importance from Table 2 is employed to determine the importance of ten sub-indicators used to build the pairwise comparison matrix. To complete the comparison matrix, our own expert judgement has been used to some extent, along with a literature review on the risks associated with oil investments, in order to assign the degrees of preference.

Table 6 illustrates the pairwise comparison matrix in which each cell represents the relative importance between sub-indicators in vertical columns with respect to the sub-indicators in horizontal rows. Previous literature on the risk of overseas oil investments is referenced to assign the preference factor needed to build a matrix (Table 6).

**Table 6:** Pairwise comparison matrix using literature review and expert judgement

Sub-indicators	GDP (billion USD dollars)	Annual inflation rate (%)	Government take (%)	Corruption index	Quality of institutions	Emerging Markets Bond Index (EMBI) Spread (%)	Proven oil reserves	Oil production	Workforce skills	Carbon dioxide emissions
GDP (billion USD dollars)	1	1/3	1/6	1/5	1/4	1/6	1/5	1/2	2	1/3
Annual Inflation Rate (%)	3	1	1/5	1/3	1/3	1/4	1/5	1/3	5	1/2
Government Take	6	5	1	2	3	2	1/2	5	7	5
Corruption Index	5	3	1/2	1	1	2	1/2	4	5	3
Quality of Institutions	4	3	1/3	1	1	2	1/2	4	5	3
Emerging Markets Bond Index (EMBI) Spread (%)	6	4	1/2	1/2	1/2	1	1/3	2	5	4
Proven Oil Reserves	5	5	2	2	2	3	1	5	7	5
Oil Production	2	3	1/5	1/4	1/4	1/2	1/5	1	2	2
Workforce Skills	1/2	1/5	1/7	1/5	1/5	1/5	1/7	1/2	1	1/5
Carbon Dioxide Emissions	3	2	1/5	1/3	1/3	1/4	1/5	1/2	5	1

In summary, the literature has indicated that fiscal regimes (e.g., tax rates, royalties) and corruption indices affect the financial decision to invest to a great extent. In developing countries, these factors are found to be more important than economic growth, annual inflation rates, and quality of institutions (Desai et al. 2001; Demirhan and Masca 2008; Levis 1979; Mathur and Singh 2013; Voyer and Beamish 2004; Yoon et al. 2021). Government bond markets and exchange rate policies in emerging economies have evolved in recent years and are being monitored as determinants for foreign participation (Andritzky 2012; del Cristo and Gómez-Puig 2017). The potential oil resources within the country have the same degree of importance as government take, since the size of the oil resource affects fiscal regime negotiations (Tang et al. 2017; Zhu et al. 2015). Many companies intend to reduce their carbon footprints; for this reason, it is expected that the energy transition may discourage investments in oil projects (Kiyar and Wittneben 2015; Plantinga and Scholtens 2021).

Equations (1) and (2) from the AHP method are applied to the pairwise comparison matrix in Table 6 to obtain the weighted index of the ten sub-indicators or the attributes used in the model (Table 7). This is justified based on the previous literature, already referenced in this paper, as well as the energy expertise of the authors, who collectively have decades of experience in industry and academia.

**Table 7:** Weight index of each sub-indicator or attribute in the model

Sub-indicators/attributes	GDP (billion USD dollars)	Annual inflation rate (%)	Government take (%)	Corruption index	Quality of institutions	Emerging markets bond index (EMBI) Spread (%)	Proven oil reserves	Oil production	Workforce skills	Carbon dioxide emissions
Criteria weights	0.026	0.041	0.208	0.136	0.128	0.107	0.234	0.052	0.019	0.049

Before continuing with the next step, the consistency index (i.e., Eq. 3) needs to be tested from Table 7. As a result, the consistency index for this model is 0.083461.

Subsequently, the consistency ratio (CR) is tested. Saaty (1977) argued that the CR (i.e., Eq. 4) needs to be less than 0.1 to be considered acceptable and to continue to the next step. Table 3 shows the random index (RI) values according to the number of attributes. For this study, the RI is 1.49 for ten sub-indicators (i.e., attributes). As a result, the CR is 0.056014, which falls well below the 0.1 threshold.

The purpose of performing the AHP method is to obtain reasonable weight coefficients of the sub-indicators, and to take into account the subjective and objective weights of the sub-indicators that will be used further in the TOPSIS method.

### Country ranking risk

A comprehensive country ranking from low- to high risk for overseas oil investments is determined using the TOPSIS method.

Firstly, data were collected for ten sub-indicators such as GDP, annual inflation rate, government take, corruption index, quality of institutions, EMBI, proven oil reserves, workforce skills, and CO_2_ emissions for the six South American countries (Table 8).

Equation (5) is applied to Table 8 to get the normalized decision matrix Rij (Table 9).

**Table 8:** Data of the ten sub-indicators for the six countries

Country	GDP (billion USD dollars)	Annual inflation rate (%)	Government Take (%)	Corruption index	Quality of institutions	Emerging markets bond index (EMBI) spread (%)	Proven oil reserves (thousand million barrels)	Oil production (Thousands of barrels per day)	Workforce skills	Carbon dioxide emissions (metric tons per capita)
Argentina	519.87	40.7	73.5	40	39.9	28.12	2.40	620.00	53.2	4.78
Bolivia	40.29	3.1	86.5	29	31.5	4.45	0.20	59.33	41.0	1.91
Brazil	1868.63	3.0	70.0	35	45.7	2.83	12.70	2877.00	39.4	2.61
Colombia	331.05	3.7	75.0	36	51.3	2.64	2.00	866.00	51.7	1.79
Ecuador	108.40	2.5	78.5	34	41.5	28.79	1.60	531.00	49.4	2.75
Peru	222.04	2.0	67.6	35	44.5	1.85	0.90	31.40	42.1	2.05

**Table 9:** Normalized decision matrix, *R*_*ij*_

*R*_*ij*_ (Matrix)										
Country	GDP (billion USD dollars)	Annual inflation rate (%)	Government take (%)	Corruption index	Quality of institutions	Emerging markets bond index (EMBI) spread (%)	Proven oil reserves (thousand million barrels)	Oil production (thousands of barrels per day)	Workforce skills	Carbon dioxide emissions (metric tons per capita)
Argentina	0.2621	0.9874	0.3978	0.4668	0.3803	0.6906	0.1817	0.1991	0.4675	0.6879
Bolivia	0.0203	0.0752	0.4681	0.3384	0.3002	0.1093	0.0151	0.0191	0.3603	0.2742
Brazil	0.9421	0.0728	0.3788	0.4084	0.4356	0.0695	0.9615	0.9238	0.3462	0.3759
Colombia	0.1669	0.0898	0.4059	0.4201	0.489	0.0648	0.1514	0.2781	0.4543	0.2575
Ecuador	0.0547	0.0607	0.4248	0.3968	0.3956	0.7071	0.1211	0.1705	0.4341	0.396
Peru	0.1119	0.0485	0.3659	0.4084	0.4242	0.0454	0.0681	0.0101	0.37	0.2952

Then, the weighted normalized decision matrix *V*_*ij*_ is obtained using the weight indices of Table 7, matrix *w*_*j*_, multiplied by Table 10 (i.e., Eq. 6).

**Table 10:** Weighted normalized decision matrix, *V*_*ij*_

*V*_*ij*_ (Matrix)										
Country	GDP (billion USD dollars)	Annual inflation rate (%)	Government take (%)	Corruption index	Quality of institutions	Emerging markets bond index (EMBI) spread (%)	Proven oil reserves (thousand million barrels)	Oil production (thousands of barrels per day)	Workforce skills	Carbon dioxide emissions (metric tons per capita)
Argentina	0.00684	0.04044	0.08268	0.06344	0.04854	0.07368	0.0426	0.01034	0.0091	0.0337
Bolivia	0.00053	0.00308	0.0973	0.04599	0.03832	0.01166	0.00355	0.00099	0.00701	0.01343
Brazil	0.0246	0.00298	0.07874	0.05551	0.05559	0.00742	0.22542	0.048	0.00674	0.01842
Colombia	0.00436	0.00368	0.08436	0.0571	0.06241	0.00692	0.0355	0.01445	0.00884	0.01262
Ecuador	0.00143	0.00248	0.0883	0.05392	0.05048	0.07544	0.0284	0.00886	0.00845	0.0194
Peru	0.00292	0.00199	0.07606	0.05551	0.05413	0.00485	0.01597	0.00052	0.0072	0.01446

Equations (7) and (8) are performed to obtain the ideal (best) value V+; and the negative ideal (worst) value V−: solutions at this stage are shown in Table 11.

Equations (9) and (10) are applied in Tables 10 and 11 to get the separation measures of each alternative (i.e., countries) from the ideal one. Table 12 shows these figures after performing this step.

**Table 11:** Ideal best and negative ideal worst of the model

Beneficial/Non-beneficial	Beneficial	Non-beneficial	Non-beneficial	Beneficial	Beneficial	Non-beneficial	Beneficial	Beneficial	Beneficial	Beneficial
Higher Value/Lower Value	Higher Value	Lower Value	Lower Value	Higher Value	Higher value	Lower value	Higher value	Higher value	Higher value	Higher value
V+ (Ideal best)	0.0246	0.00199	0.07606	0.06344	0.06241	0.00485	0.22542	0.048	0.0091	0.0337
V− (Negative ideal worst)	0.00053	0.04044	0.0973	0.04599	0.03832	0.07544	0.00355	0.00052	0.00674	0.01262

**Table 12:** Separation measure from the ideal (best) and ideal (worst) solutions

Country	Si+	Si−
Argentina	0.204	0.052
Bolivia	0.232	0.074
Brazil	0.019	0.243
Colombia	0.195	0.09
Ecuador	0.216	0.05
Peru	0.217	0.086

Finally, Eq. (11) is applied to Table 12 to obtain the Pi values (i.e., performance score values), which indicate the most preferred and least preferred feasible solution. Table 13 depicts the Pi figures and ranking of each country, assigning the best position (i.e., low investment risk) to countries that have high Pi values.

**Table 13:** TOPSIS arrangement for six countries

TOPSIS arrangement		
Country	Pi	Rank
Brazil	0.927	1°
Colombia	0.316	2°
Peru	0.284	3°
Bolivia	0.242	4°
Argentina	0.204	5°
Ecuador	0.187	6°

## Results and analysis

This section highlights the main findings obtained after applying the AHP and TOPSIS methods. For a better understanding, it has been subdivided into three sections: results of AHP, results of TOPSIS, and analysis.

### Results of AHP

Table 14 shows the results of the assigned values to complete the judgment matrix for the ten sub-indicators, where it is shown that the majority of these values for each risk category are similar. Only for the risk category ‘Oil Resource Potential’ are there great differences between the values assigned for the sub-indicators, ‘Proven Oil Reserves’ and ‘Oil Production’.

**Table 14:**
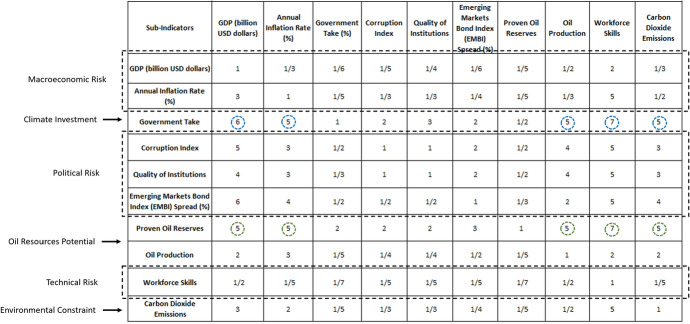
Judgment matrix in the risk of overseas oil assessments

The blue and green circles in Table 14 indicate that the ‘Proven Oil Reserves’ and ‘Government Take’ have high-scale values of importance with respect to the other sub-indicators. On the other hand, ‘Workforce Skills’ has the lowest assigned values with respect to the other sub-indicators. Table 14 shows the judgment matrix for the ten sub-indicators or attributes.

The analysis is in concordance with the results shown in Table 15, where each weight index for ‘Proven Oil Reserves’ and ‘Government Take' are in the first and second place (based on the comprehensive weight index), while the ‘Workforce Skills’ is in the tenth position. Therefore, five sub-indicators—proven oil reserves, government take, corruption index, quality institutions and EMBI (following that order)—are the most critical sub-indicators or attributes due to their high comprehensive weight values. For this reason, IOCs should prioritize analysis of these risk factors before investing in the upstream oil sector (Duan et al. 2018).

**Table 15:** Arrangement of the comprehensive weight index for the ten sub-indicators

Sub-indicators/attributes	Proven oil reserves	Government take (%)	Corruption index	Quality of institutions	Emerging markets bond index (EMBI) spread (%)	Oil production	Carbon dioxide emissions	Annual inflation rate (%)	GDP (billion USD dollars)	Workforce skills
Criteria weights	0.234	0.208	0.136	0.128	0.107	0.052	0.049	0.041	0.02	0.019
Arrangement	1°	2°	3°	4°	5°	6°	7°	8°	9°	10°

### Results of TOPSIS

As shown in Table 13, the risk order (from low- to high risk) for overseas oil investments in South America is as follows: Brazil, Colombia, Peru, Bolivia, Argentina and Ecuador.

Regarding the country-ranking based on the TOPSIS method, Brazil has the second-largest proven oil reserves in South America after Venezuela; however, Venezuela is not included in this study due to its economic and political instability and the lack of available data. In addition, Brazil’s ‘Government Take’ is among the lowest in this region (see Table 8), which helps it to obtain the highest score in the performance score value (Pi) and thus places the country at the lowest risk level with regard to overseas oil investments (see Table 13).

Colombia and Peru also present lower risks for FDI within the upstream oil sector because Colombia has the best quality of institutions (see Table 8), and the EMBI spread in Peru is significantly lower, which means it is a good place to invest with low risk. Conversely, Argentina and Ecuador have higher risks due to the high inflation rate and high EMBI spread, respectively (see Table 8), which makes these countries uncertain prospects for IOCs. The World Bank (2020) in the 17th edition of the report ‘Doing Business’ evaluated the foreign investments in 190 economies. The findings were in concordance with the results obtained from TOPSIS, showing the best performance was that of Colombia and Peru; on the other side of the coin, the worst performance was that of Argentina and Ecuador.

Although the risk assessment in this study is taken into consideration based on the outcomes of the AHP and TOPSIS methods, a more detailed evaluation of the economic, financial, and environmental benefits of overseas oil investment, the energy return on investment (EROI) calculation, and other factors that affect the investment decision in the upstream oil sector were not considered in the analysis. Therefore, IOCs cannot rely solely on the country-ranking risk shown in Table 13 to make the best possible decision. The optimal oil investment decision-making is the result of considering a wide range of factors associated with the upstream oil industry. Nevertheless, the proposed country ranking can serve as a tool to provide complementary and important oil investment information to the IOCs.

### Analysis

The development and promotion of EIs is expected to contribute to the economic growth of South America after the COVID-19 crisis. However, volatile oil prices and the challenges to exploit oil resources (such as the pre-salt resources in Brazil, shale oil in Argentina, and remote reserves in the Amazon rain forest between Colombia, Ecuador and Peru) requires substantial economic investments and human resources. These oil prospects are accompanied by a variety of risk factors, so it is important to be able to identify and evaluate them before investing. South America’s oil resource potential represents 18.7% of the world’s total proven oil reserves and provides 6.5% of oil production worldwide (BP 2020). Table 16 shows the figures of oil reserves and production in 2019 for the six South American countries analyzed. Reserves to production ratios, a proxy for life expectancies, are also included.

**Table 16:** Oil reserves and production in selected South American countries, 2019

Country	Proven Oil reserves (thousand million barrels)	Oil production (thousands of barrels per day)	Reserves to production ratio (years)
Argentina	2.4	620	10.5
Bolivia	0.2	59	9.3
Brazil	12.7	2877	12.1
Colombia	2.0	886	6.1
Ecuador	1.6	531	8.4
Peru	0.9	142	16.5

Source: BP (2020); IEA (2020)

According to Table 16, Brazil has by far the highest proven oil reserves compared to the rest of the countries. It also has the highest oil production by a sizeable margin.

In South America, the state-owned oil companies have the largest participation within the petroleum industry through Production Sharing Contracts (PSCs) and Risk Service Contracts (RSCs). The state-owned companies dedicated to exploration and production (E&P) are PDVSA (Venezuela), PETROBRAS (Brazil), ECOPETROL (Colombia), YPFB (Bolivia), PETROAMAZONAS (Ecuador) and YPF (Argentina).

Although Venezuela is an oil-rich country (IEA 2020), as referred to earlier, its political instability gives it the highest levels of corruption, debt, hyperinflation and EMBI spread. Moreover, the high proportion of its ‘Government Take’ makes the country unfavorable for petroleum investment (Rodriguez et al. 2012).

## Policy recommendations

This section suggests policy recommendations to make the region more competitive in promoting its petroleum resources.

Strengthening the perception of fiscal stability and credibility is crucial to attracting IOCs without deterring foreign oil investments (Daniel et al. 2017). The unprecedented challenges for petroleum projects during the recent oil downturn, caused by geopolitical issues between OPEC+ members and the COVID-19 crisis, suggest that fiscal regimes and policy instruments must be restructured to support and promote the oil industry. For enhanced cooperation, it is important to consider the perspectives of both parties involved in upstream oil activities.

- IOCs are recommended to use the results obtained in this research as an additional tool that can complement other types of detailed analysis on the benefits of investing in a given country.
- Focusing on evaluating the first five indicators—proven oil reserves, government take, corruption index, quality of institutions and EMBI—serves the IOC to have a better investment perspective; however, it is recommended that additional economic and financial indicators be considered to improve this perspective.
- The investment landscape is directly influenced by the potential of oil resources and government take in a given country. Thus, it is recommended that IOCs analyze in detail the political perception of the country over the estimated life of the oil project.

### Government perspectives

The host government will always seek to maximize oil resource revenues. However, to remain an attractive destination for investment, it needs to create equitable and sustainable fiscal regimes where the risk and reward is shared between both parties. This leads to reduced financial risk and at least a minimum required return after-tax cash flow for the investors or IOCs.

The host government must be able to design, evaluate, and reform macro-fiscal policies that aim to attract FDI, but these policies should be oriented to:

- Economic diversification to support future prosperity, which will make the economy more resilient to non-renewable commodity downturns.
- Fiscal policies that are flexible to exchange rates and less procyclical.
- Consideration and mitigation of commodity price volatility.
- Improved procedures for environmental impact studies.

### Investor perspectives

Foreign investors look for fiscal, legal and administrative guarantees (e.g., contract and property rights) that are based on equitable and neutral regimes that help to secure and not distort the investment decision through the life of the project.

Investors expect that the macro-fiscal framework (i.e., the government regulation) should be simple to understand to meet all the commitments with the host government. They also expect that the negotiation of the oil contracts should be transparent, thus generating certainty and stability for both parties.

IOCs furthermore seek a relatively developed oil industry in the country where they plan to invest, so they can obtain technical information on oil resources and the environment.

## Conclusions

This study develops a comprehensive country ranking, from low- to high risk, of overseas oil investments, which helps to have a better understanding about competitiveness in South America. The conclusions are the following:

- Before any investment decision is made, IOCs need to be aware of the first five sub-indicators in the following order (based on their criteria weight from the AHP methodology—see Table 15): ‘Proven Oil Reserves’, ‘Government Take’, ‘Corruption Index’, ‘Quality of Institutions’ and ‘EMBI Spread’.
- Government Take has the second highest weight indices of importance from the AHP method. Hence, FDIs are mainly driven by corporate taxation and controlled by factors such as economic growth and exchange rate volatility, which generate positive and negative impacts on international capital flows (Kiyota and Urata 2004).
- The main outcome obtained from the TOPSIS methodology is the comprehensive country risk ranking for upstream oil investments in South America. From low- to high risk, the ranking is as follows: Brazil, Colombia, Peru, Bolivia, Argentina and Ecuador (Table 13).
- Success in attracting IOCs does not depend solely on factors like significant petroleum resources, large domestic markets, and inexpensive labor. If a country can make optimal adjustments to its investment regime, it will increase its ability to successfully attract foreign capital.

Notwithstanding the contribution of this paper, there are inevitable shortcomings in this study and a need for further research. The work focuses its analysis on data gathered between 2019 and 2020 (i.e., before the COVID-19 outbreak) for the sub-indicators of six oil-producing countries in South America. It excludes countries that have high economic and political instability, limited oil resource potential or are net importers of oil. Examples that fall into these categories include Venezuela, Chile, Paraguay, Uruguay, Suriname and Guyana. The study employs ten relevant sub-indicators connected to the upstream oil industry, which were obtained from the literature review and our own expert judgement; however, there are certainly sub-indicators not included, such as exchange rate volatility. The shortage of available data to extend the time horizon of the analysis is one of the main constraints of this study. For this reason, the work follows a deterministic model that focuses on data before COVID-19. While the findings indicate the need for business-friendly environments to attract foreign oil investors (e.g., through the reassessment of fiscal regimes by host countries), the study does not conduct a deep analysis about how the host country should modify its petroleum fiscal regime and other policy instruments. For this reason, the impacts caused by the pandemic on foreign oil investments, and the impacts of FDI flows on corporate taxation (especially in countries with high government take) should be analyzed in future research on the region.

## Data Availability

All data generated or analysed during this study are included in this published article.
